# Head and neck tumor cells treated with hypofractionated irradiation die via apoptosis and are better taken up by M1-like macrophages

**DOI:** 10.1007/s00066-021-01856-4

**Published:** 2021-10-19

**Authors:** Hanna Wedekind, Kristina Walz, Mayte Buchbender, Thorsten Rieckmann, Erwin Strasser, Fridolin Grottker, Rainer Fietkau, Benjamin Frey, Udo S Gaipl, Michael Rückert

**Affiliations:** 1 Translational Radiobiology, Department of Radiation Oncology, Friedrich-Alexander-Universität Erlangen-Nürnberg, Universitätsklinikum Erlangen, Erlangen, Germany; 2 Department of Radiation Oncology, Friedrich-Alexander-Universität Erlangen-Nürnberg, Universitätsklinikum Erlangen, Erlangen, Germany; 3 Department of Oral and Maxillofacial Surgery, Friedrich-Alexander-Universität Erlangen-Nürnberg, Universitätsklinikum Erlangen, Erlangen, Germany; 4 Department of Radiotherapy and Radiation Oncology, University Medical Center Hamburg Eppendorf, Hamburg, Germany; 5 Department of Otolaryngology and Head and Neck Surgery, University Medical Center Hamburg Eppendorf, Hamburg, Germany; 6 Department of Transfusion Medicine and Hemostaseology, Friedrich-Alexander-Universität Erlangen-Nürnberg, Universitätsklinikum Erlangen, Erlangen, Germany

**Keywords:** Head and neck cancer, Macrophage polarization, Phagocytosis, Radiotherapy, Human papilloma virus

## Abstract

**Purpose:**

The incidence of head and neck squamous cell carcinomas (HNSCC) is increasing worldwide, especially when triggered by the human papilloma virus (HPV). Radiotherapy has immune-modulatory properties, but the role of macrophages present in HNSCC and having contact with irradiated tumor cells remains unclear. The influence of irradiated (2 × 5Gy) HNSCC cells on the (re-)polarization and phagocytosis of human macrophages, either non-polarized or with a more M1 or M2 phenotype, was therefore investigated.

**Methods:**

Human monocytes were differentiated with the hematopoietic growth factors M‑CSF (m) or GM-CSF (g) and additionally pre-polarized with either interleukin (IL)-4 and IL-10 or interferon (IFN)-γ and lipopolysaccharides (LPS), respectively. Subsequently, they were added to previously irradiated (2 × 5Gy) and mock-treated HPV-positive (UD-SCC-2) and HPV-negative (Cal33) HNSCC cells including their supernatants.

**Results:**

The HNSCC cells treated with hypofractionated irradiation died via apoptosis and were strongly phagocytosed by M0m and M2 macrophages. M0g and M1 macrophages phagocytosed the tumor cells to a lesser extent. Irradiated HNSCC cells were better phagocytosed by M1 macrophages compared to mock-treated controls. The polarization status of the macrophages was not significantly changed, except for the expression of CD206 on M2 macrophages, which was reduced after phagocytosis of irradiated HPV-negative cells. Further, a significant increase in the uptake of irradiated HPV-positive cells by M0g macrophages when compared to HPV-negative cells was observed.

**Conclusion:**

HNSCC cells treated with hypofractionated irradiation foster phagocytosis by anti-tumorigenic M1 macrophages. The data provide the first evidence on the impact of the HPV status of HNSCC cells on the modulation of the macrophage response to irradiated tumor cells.

## Introduction

Head and neck squamous cell carcinoma (HNSCC) is the sixth most common cancer worldwide, with more than 890,000 new cases every year [1]. The mortality rate has not decreased for decades, and the 5‑year survival rate is under 70% [2]. Not only the consumption of alcohol and tobacco [3, 4] but also an infection with the human papilloma virus (HPV) are strongly associated with HNSCC. The incidence of HPV-driven head and neck tumors is rapidly increasing [5, 6]. Apart from surgery, adjuvant or primary treatment with radiotherapy (RT) is common. Patients with solid tumors receive RT in about 60% of the cases during the course of their disease [7]. It should be emphasized that ionizing radiation not only has a direct influence on the irradiated tumor cells, but also has a broader systemic effect through the induction of immunogenic cell death, which has a modulating and stimulating effect on the immune system, leading in the ideal case to antitumoral immune responses [8–14].

The immune system plays a crucial role in the development of tumors [15–18], which becomes clear in the interaction between tumor cells and the tumor microenvironment (TME; [19, 20]). The latter is composed of many different cells and molecules, such as tumor cells, immune cells, tumor-associated fibroblasts, the vascular network, cytokines, growth factors, chemokines, or enzymes. The cancer–immune cell crosstalk is a dynamic process that is influenced by multiple factors and determines whether a pro- or anti-inflammatory environment is established at the tumor site [21]. A major part of the immune compartment of solid tumors comprises tumor-associated macrophages (TAMs; [22]).

Macrophages develop and differentiate from monocytes via the hematopoietic growth factors macrophage-stimulating factor (M-CSF; [23]) and granulocyte macrophage-stimulating factor (GM-CSF; [24]) and can be categorized as CD11b-positive cells [25]. The differentiated M0 macrophages (M0-MΦ) are able to polarize into two key phenotypes depending on the existing microenvironment and signal molecules surrounding them [26]. The pro-inflammatory M1 macrophages (M1-MΦ), called “classically activated macrophages” [27–32], are polarized by lipopolysaccharide (LPS) and interferon gamma (IFN‑γ; [33–36]) and are characterized by a high expression of the MHC (class II) cell surface receptor HLA-DR [25] and the co-stimulatory activation marker CD80 [37, 38]. The anti-inflammatory M2 macrophages (M2-MΦ), called “alternatively activated macrophages”, can be polarized by the cytokines IL‑4 and IL-10 [32, 38–41] and are characterized by a high expression of the scavenger receptor CD163 [38, 40, 42] and the mannose receptor CD206 [25, 38, 43–45]. They are considered as immunosuppressive macrophages [18, 27–29, 46–51]. Nevertheless, macrophages have a high plasticity, being able to switch from one phenotype to another, which means they have the ability to re-polarize depending on the microenvironment and on specific stimuli they are exposed to [29, 52–54]. Furthermore, fully polarized M1- and M2-MΦ are the extremes of a continuum of functional states.

Tumor-associated macrophages can act in pro- or anti-inflammatory ways [22, 55, 56], but are predominantly considered as protumoral M2-like macrophages and play a key role in regulating inflammation, angiogenesis, cancer cell migration, tissue remodeling, and tumor progression [22, 57–60]. The mutual interaction between macrophages and HNSCC cells triggers a polarization of TAMs toward M2-MΦ as well as tumor invasion and angiogenesis [61]. Furthermore, one of the primary functions of macrophages and central mechanisms by which TAMs can affect cancer progression is through phagocytosis of pathogens and abnormal cells including tumor cells [22, 62–64]. Phagocytosis is essential for maintaining normal homeostasis and healthy tissue, and it is a therapeutic target for a wide range of clinical applications [65–67].

According to Okubo et al. [68], RT leads to vascular damage and hypoxia of the tumor, which in turn leads to migration of CD11b-positive cells, polarizing into M2-MΦ [69]. There is a well-known correlation between a high infiltration of M2-MΦ and tumor progression of HNSCC, leading to an overall poorer prognosis [46, 57, 70]. In addition, it should be emphasized that HPV-positive patients generally show better responses to RT, and thus have a better prognosis and overall survival than HPV-negative patients [5, 6, 71–74]. This is most likely due to immune responses triggered by HPV infections, which may be further enhanced by RT [75].

Considering that TAMs contribute to the formation of an immunosuppressed state within the TME, one of the therapeutic strategies targeting TAMs is reeducating TAMs to an antitumor phenotype, such as promoting the phagocytosis ability of macrophages [22, 76].

Our objectives were therefore to investigate the influence of irradiated HNSCC cells on the (re-)polarization and the phagocytosis of human macrophages, either being non-polarized or having a more M1 or M2 phenotype, by bringing them into direct cell-to-cell contact with each other. One HPV-positive and one HPV-negative HNSCC cell line was used for the examinations.

## Materials and methods

### Cell lines and cell culture

The head and neck tumor cell lines Cal33 (cal33; HPV-negative) and UD-SCC‑2 (UD2; HPV-positive) were cultured in DMEM medium (Pan Biotech) containing 10% fetal bovine serum (FBS, Sigma-Aldrich) and 1% penicillin/streptomycin (PenStrep, GIBCO Life Technologies, Thermo Fisher Scientific, Waltham, MA, USA). Monocytes from healthy human donors (isolation procedure described below) were cultured in RPMI1640 medium (Sigma-Aldrich, St. Louis, MO, USA) containing 10% FBS and 1% PenStrep. All cells were incubated under standard conditions at 37 °C, 5% CO_2_, and 95% humidity (Incubator RBP 6220, Hereus Instruments, Hanau, Germany).

### Monocyte isolation

Peripheral blood mononuclear cells (PBMC) were isolated from leukoreduction system chambers (LRSC) of healthy, anonymous donors having undergone a strict health check by the Transfusion Medicine and Hemostaseology Department of the Universitätsklinikum Erlangen, Germany. The permission to use this LRSC was given by the ethics committee of the Friedrich-Alexander-Universität Erlangen-Nürnberg (ethical approval no. 180_13 B and 48_19 B) according to the rules of the Declaration of Helsinki in its current form. All PBMC were isolated by density gradient centrifugation and subsequently monocytes were separated by CD14+ selection using magnetic cell sorting (MACS; Miltenyi Biotec, Bergisch Gladbach, Germany) following the manufacturer’s instructions. The selected naive monocytes were seeded in six-well plates with a density of 1.5 × 10^6^ cells per well in RPMI1640 medium (Sigma-Aldrich) containing 10% FBS and 1% PenStrep.

### Macrophage differentiation and pre-polarization

Monocytes were differentiated to macrophages by adding either M‑CSF (100 µg/ml, Miltenyi Biotec, Peprotech) for M0m-MΦ or GM-CSF (40 µg/ml, Miltenyi Biotec) for M0g-MΦ to the monocyte culture on day 0, 2, and 5. On day 5, cells were washed and fresh medium was added. All M0g-MΦ were additionally polarized to M1-MΦ by adding IFN‑γ (1 mg/ml, R&D Systems, Minneapolis, MN, USA) and LPS (5 mg/ml, Sigma-Aldrich). M0m-MΦ were additionally polarized to M2-MΦ by adding IL‑4 (recombinant, human, 50 µg/ml, Miltenyi Biotec) and IL-10 (recombinant, human, 100 µg/ml, Miltenyi Biotec).

### Tumor cell irradiation

Head and neck tumor cells were treated with hypofractionated RT (HFX-RT) with 5 Gy of X‑rays using an X‑ray tube in a lead shielding chamber (X-Ray generator Isovolt Titan, GE Inspection Technologies, Hürth, Germany) 24 and 48 h after seeding or they were left untreated functioning as mock-treated controls.

### Cell death analysis

Cell death was analyzed 24 h after the last irradiation of the tumor cells by FITC-labeled AnnexinA5 (AxV, Geneart, Life Technologies) and propidium iodide (PI, Sigma-Aldrich) staining. Analyses were performed with the CytoFLEX S flow cytometer (Beckmann Coulter, Brea, CA, USA) and the Kaluza software. AxV^-^, PI^-^ cells were defined as viable, AxV^+^, PI^-^ as apoptotic, and AxV^+^, PI^+^ as necrotic cells.

### Phagocytosis assay

Previously irradiated and non-irradiated tumor cells were stained with the cytoplasmic membrane dye CellBrite (Biotium, Hayward, CA, USA), following the instructions obtained by the producer. After the staining, the tumor cells were resuspended in their own corresponding supernatant, in which they had been incubated and treated for the previous 3 days. Then the stained tumor cells were added to the differentiated and pre-polarized macrophages at a ratio of 3:1 (6 × 10^5^ tumor cells per 2 × 10^5^ macrophages) and incubated at 37 °C for 2h. As a negative control, cells were additionally stored at 4 °C immediately after the addition of the tumor cells to the macrophages.

Subsequently, 2 h after the addition, all (living and dead) cells were harvested by using a cell scraper, scraping off the adherent cells from the bottom of the wells and rinsing them with PBS (4 °C). For the analyses of the phagocytosis ability, the cells were stained with the viability dye Zombie NIR (BioLegend, Koblenz, Germany) and anti-human CD11b (BV635, BioLegend) to distinguish macrophages from tumor cells. Analyses were performed with the CytoFlex S flow cytometer and the Kaluza software. The gating strategy is displayed in Fig. 3b.

### Polarization analysis

To analyze the phenotype of the macrophages, the stained irradiated and mock-treated tumor cells (described above) including their corresponding supernatants were additionally added to the differentiated and pre-polarized macrophages at the same ratio of 3:1 (6 × 10^5^ tumor cells per 2 × 10^5^ macrophages) and were incubated at 37 °C for 24 h. As a negative control, macrophages of each condition were left without the addition of any tumor cells (referred to as “w/o”).

All cells were harvested 24 h after the addition, as described above. The following dyes and antibodies conjugated to the indicated fluorochromes were used for the stainings: Zombie NIR (BioLegend), CD11b (BV635, BioLegend); CD80 (PE-Cy7, BioLegend); HLA-DR (KO, Beckman Coulter); CD163 (PE-Dazzle, BioLegend); and CD206 (APC, BioLegend). In addition, cells were only stained with Zombie NIR and CD11b. Analyses were performed with the CytoFlex S flow cytometer and the Kaluza software. Background correction was carried out by subtracting the median fluorescence intensity of the Zombie NIR and CD11b staining from the complete staining.

### Statistical analysis

Statistical analyses were performed with the GraphPad Prism 8 software. Unless indicated otherwise, a Kruskal–Wallis test was calculated to compare all treatment groups against the negative control with Dunn’s correction for multiple testing and a Mann–Whitney *U* test to compare two treatment groups. Significance is indicated as follows: *p < 0.05, **p < 0.01, ***p < 0.001.

## Results

### In vitro differentiated and (pre-)polarized macrophages show characteristic M1 and M2 marker expression

**Fig. 1:**
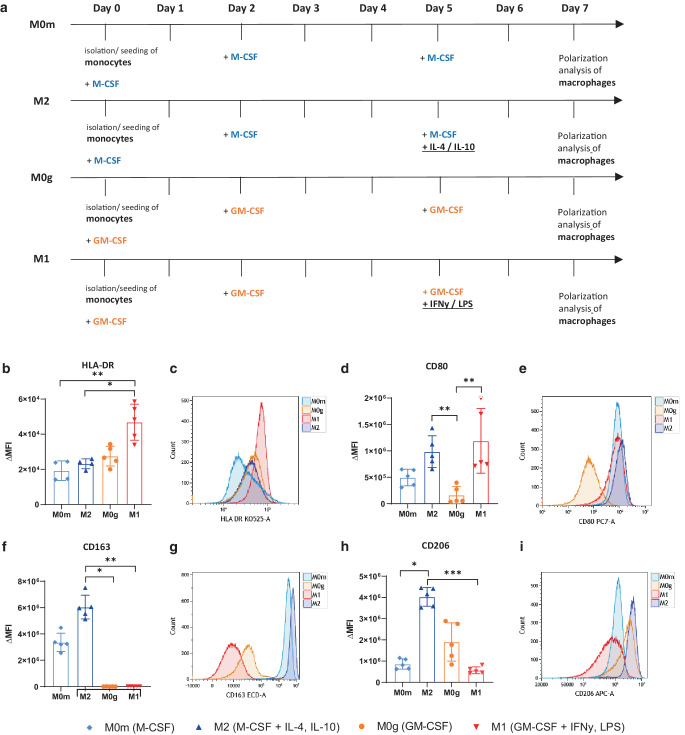
Human in vitro differentiated macrophages show the characteristic M1 and M2 marker expression after polarization with respective cytokines. **a** Timeline shows the different treatment schedules of the naive human monocytes, differentiating and (pre‑)polarizing into the four phenotypes of the macrophages M0m‑, M0g‑, M1-, and M2-MΦ, which were used in the experiments. On day 0, PBMCs were isolated from concentrated human blood by density gradient centrifugation and monocytes were isolated by CD14+ MACS separation. On day 0, 2, and 5 the growth factors M‑CSF for M0m- and M2-MΦ and GM-CSF for M0g- and M1-MΦ were added to the medium. On day 5 the medium was renewed and the cytokines IFN‑γ and LPS for M1-MΦ and IL‑4 and IL-10 for M2-MΦ were additionally added. For the polarization analysis of the macrophages, all cells were stained with the viability stain Zombie NIR and the antibodies CD11b, CD80, HLA-DR, CD163, and CD206 (**b–i**). Background correction for the respective marker expression was performed by subtracting the median fluorescence intensity of the Zombie NIR and CD11b staining from the complete staining. Background corrected (ΔMFI) expression data for HLA-DR (**b**), CD80 (**d**), CD163 (**f**), and CD206 (**h**) are presented as median with interquartile range and additionally representative data for each marker (**c, e, g, i**) are depicted. Statistical analysis was performed with the Kruskal–Wallis test and Dunn’s correction for multiple testing comparing all four conditions with each other. Significance is indicated as: * *p* < 0.05, ** *p* < 0.01, ***p < 0.001

To investigate the phagocytosis and subsequent (re-)polarization of differentiated and pre-polarized macrophages in vitro, human monocytes were differentiated for 7 days with the hematopoietic growth factors M‑CSF or GM-CSF and pre-polarized during the 48 h with either IL‑4 and IL-10 or IFN‑γ and LPS, respectively (Fig. 1a). The differentiation and (pre‑)polarization resulted in four different phenotypes of macrophages that were consecutively used for the examinations: M0m‑, M0g‑, M1-, and M2-MΦ. These were subsequently analyzed for their expression of HLA-DR, CD80, CD163, and CD206 (Fig. 1b–i).

The M1-marker HLA-DR (Fig. 1b and c) was expressed on all four phenotypes, but was expressed significantly higher on M1- compared to M0m- and M2-MΦ. The expression of the activation marker CD80 (Fig. 1d and e) was not only significantly higher on M1-, but also on M2-MΦ compared to M0g-MΦ, and it was also slightly up-regulated on M0m-MΦ. M2-MΦ expressed the highest levels of the typical M2-markers CD163 (Fig. 1f and g) and CD206 (Fig. 1h and i). CD206 showed significantly higher expression on M2-MΦ compared to M1- and M0m-MΦ. Whereas CD163 was absent on all GM-CSF-differentiated cells, CD206 was up-regulated on M0g-MΦ too. These results confirm that macrophages of high plasticity and the key immune phenotypes were available for the subsequent analyses.

### Hypofractionated irradiation of HNSCC cells induces predominantly apoptotic tumor cell death

**Fig. 2:**
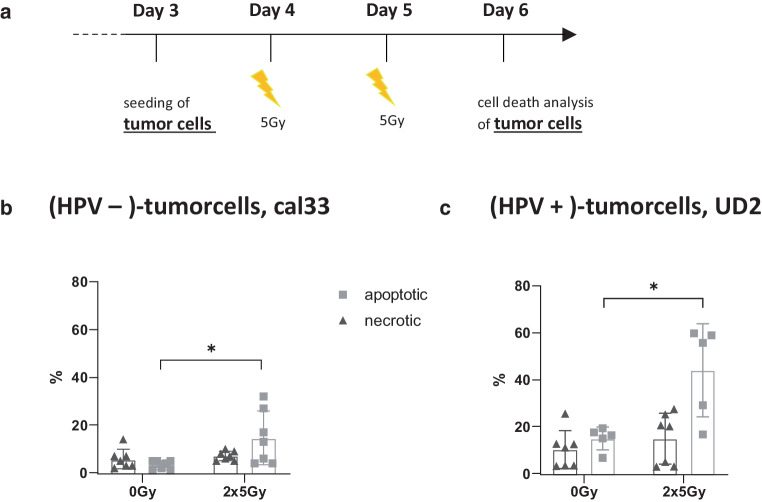
Hypofractionated irradiation induces predominantly apoptosis in HNSCC cells. **a** Timeline shows the treatment of the HNSCC cells starting on day 3 by seeding the cells and incubating them for the following 3 days at standard culture conditions. The tumor cells were irradiated with 5 Gy on day 4 and 5 (2 × 5Gy). The control group (mock-treated) was left untreated (0 Gy). On day 6, 24 h after the last irradiation, cell death analysis of the tumor cells was performed by FITC-labeled AnnexinA5 (AxV) and propidium iodide (PI) staining. Cell death is represented by the necrotic and apoptotic cell population of the HPV-negative HNSCC cell line cal33 (**b**) and the HPV-positive HNSCC cell line UD2 (**c**). Necrotic cells were defined as PI+, apoptotic cells as AxV+ and PI−. Statistical analysis was carried out with the Mann–Whitney *U* test. Significance is indicated as: * *p* < 0.05. Data are presented as mean with SD

Irradiation with 2 × 5 Gy significantly increased apoptosis in the HPV-negative HNSCC cell line cal33 and in the HPV-positive HNSCC cell line UD2 as early as 24 h after the last irradiation (Fig. 2b and c). By contrast, necrosis was not significantly altered by hypofractionated irradiation of the examined HNSCC tumor cells at the early time point analyzed.

### Only M1 macrophages show increased phagocytosis of irradiated HNSCC cells compared to mock-treated ones

**Fig. 3:**
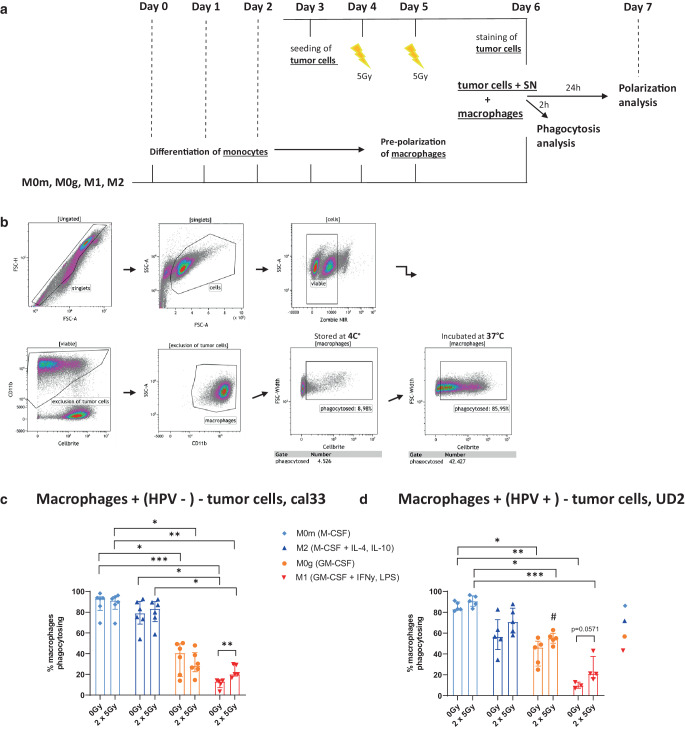
Irradiated HNSCC cells are better taken up by M1 macrophages compared to mock-treated ones. **a** Timeline shows the simultaneous treatment of the HNSCC cells and the monocytes/macrophages. On day 6 the tumor cells were first stained with the cytoplasmic membrane dye CellBrite and subsequently added to the differentiated and pre-polarized macrophages at a ratio of 3:1 (6 × 10^5^ tumor cells per 2 × 10^5^ macrophages) and incubated at 37 °C in the corresponding supernatants (SN) in which the tumor cells were treated for the previous 3 days. After 2 h, all cells were harvested and stained with the viability stain Zombie NIR and CD11b to distinguish between the two cell types. As a control group, macrophages and tumor cells were incubated at 4 °C. The gating strategy of the phagocytosis analysis is shown in **b**. After pre-gating on singlets (FSC-A/FSC-H) and viable cells based on morphological properties (FSC-A/SSC-A) and live/dead stain (Zombie NIR), macrophages were identified by CD11b expression. The phagocytosis rate was determined by the percentage of CellBrite-positive macrophages. Data for the uptake of the 2 × 5Gy-irradiated and mock-treated (0 Gy) HPV-negative HNSCC cell line cal33 (**c**) and the HPV-positive HNSCC cell line UD2 (**d**) are presented as median with interquartile range. Statistical analysis was performed with the Kruskal–Wallis test and Dunn’s correction for multiple testing comparing all “0 Gy” and all “2 × 5 Gy” of all four phenotypes with each other, and with the Mann–Whitney *U* test comparing “0 Gy” with “2 × 5 Gy” within each phenotype. Additionally, the Mann–Whitney *U *test was used to compare the influence of the HPV status on the uptake of tumor cells through the respective macrophage subtypes and treatment modalities (# *p* < 0.01). Significance is indicated as: * *p* < 0.05, ** *p* < 0.01, *** *p* < 0.001

To examine whether the different macrophage subsets take-up HNSCC cells differently dependent on their previous irradiation and HPV-status, the macrophages were brought together in direct cell-to-cell contact with the differently treated head and neck tumor cells and their corresponding supernatants for 2 h and phagocytosis was determined by flow cytometry (Fig. 3a and b). Macrophages that were differentiated with M‑CSF (M0m- and M2-MΦ) were characterized by significantly higher phagocytosis rates compared to GM-CSF-differentiated ones (M0g- and M1-MΦ; Fig. 3c and d). However, no differences in the uptake of irradiated tumor cells compared to mock-treated ones were observed. By contrast, M1-MΦ took up irradiated HNSCC tumor cells irrespectively of their HPV status better than non-irradiated mock-treated ones (Fig. 3c and d). The irradiated HPV-positive tumor cells (Fig. 3d) were taken up significantly better by M0g0MΦ than the irradiated HPV-negative tumor cells (Fig. 3c).

### Polarization of macrophages is hardly affected by addition of HNSCC cells including their SN regardless of previous treatment and HPV status

**Fig. 4:**
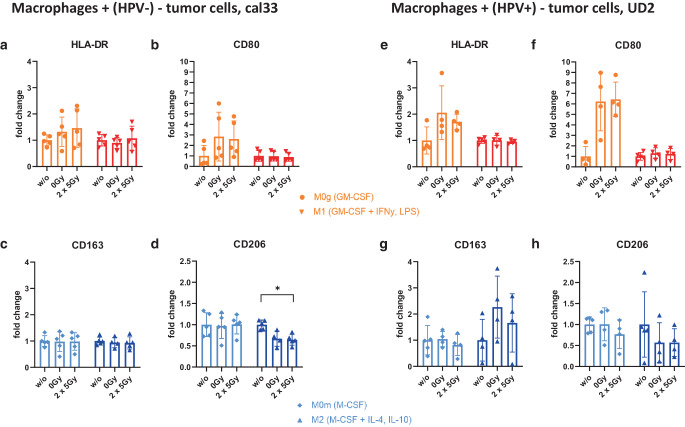
Macrophages are not (re-)polarized upon the uptake of HPV-positive or HPV-negative HNSCC cells including their supernatants, but CD206 is downregulated on M2 macrophages. For the analysis of the (re-)polarization of the macrophages after the addition of irradiated (2 × 5 Gy) and mock-treated (0 Gy) HPV-negative (cal33; **a–d**) and HPV-positive (UD2; **e–h**) HNSCC cells, after 24 h macrophages were stained with the viability stain Zombie NIR and the antibodies for CD11b, HLA-DR, CD80, CD163, and CD206. Macrophages without the addition of any tumor cells are referred to as “w/o,” and used as control groups. Background correction for the respective marker expression was performed by subtracting the median fluorescence intensity of the Zombie NIR and CD11b staining from the complete staining. The fold change in expression of the markers HLA-DR (**a, e**), CD80 (**b, f**), CD163 (**c, g**), and CD206 (**d, h**) is presented in relation to the “w/o” control. Statistical analysis was performed with the Kruskal–Wallis test and Dunn’s correction for multiple testing comparing “w/o,” “0 Gy,” and “2 × 5 Gy” with each other within each phenotype. Significance is indicated as: * *p* < 0.05

The analysis of the polarization and re-polarization of the macrophages after the addition of mock-treated and irradiated tumor cells, respectively, is shown in Fig. 4. To include the stimulus of the tumor cells themselves as well as potential soluble factors, the tumor cells were added to the macrophages resuspended in the SN collected after the respective treatment.

The addition of both HNSCC cell lines including their corresponding SNs, irrespective of their treatment (0 Gy- vs. 2 × 5 Gy-irradiation), did not affect the polarization of the macrophages (Fig. 4a–c, e–g)**,** except for the expression of CD206. Its expression tended to be lower after contact of M2-MΦ with both mock-treated and irradiated HNSCC cells (Fig. 4d and h). In the case of the HPV-negative cell line (cal33), the expression of CD206 was even significantly decreased after contact with irradiated tumor cells (Fig. 4d).

## Discussion

Macrophages are major components of the TME in many solid tumors, such as HNSCC. Considering that TAMs contribute to the formation of an immunosuppressed state within the TME, one of the therapeutic strategies targeting TAMs is reeducating TAMs to an antitumor phenotype, such as promoting the primary function of macrophages: their phagocytosis ability [22, 76]. Therefore, macrophages represent promising targets for anticancer therapy in humans. Surgery, RT, or both, have been used for decades to achieve locoregional control. Considering that approximately 60% of patients with solid tumors such as HNSCC receive RT in the course of their disease [7], it is mandatory to have a better understanding of how RT treatment affects the function and phenotype of macrophages being in contact with irradiated HNSCC cells.

In our in vitro study we succeeded in establishing four stable macrophage phenotypes by differentiating and pre-polarizing them out of monocytes of healthy human donors (Fig. 1a). The MHC-II-complex HLA-DR was found to be the most applicable one for identifying M1-MΦ (Fig. 1b and c), whereas the scavenger receptor CD163 (Fig. 1f and g) and the mannose receptor CD206 (Fig. 1h and i) both proved to be suitable forM2-MΦ. With those established phenotypes it was possible to set up phagocytosis assays and analyses of re-polarization after bringing those macrophages in direct cell-to-cell contact with the HNSCC cells and their SN.

Considering that not only the consumption of alcohol and tobacco, but also an infection with HPV is strongly associated with HNSCC [1] and that HPV-associated HNSCC have split off as an independent subgroup in recent years [77], both an HPV-negative and an HPV-positive HNSCC cell line was included in the examinations. Approximately 20–25% of affected patients are HPV-positive and among them the HPV subtype 16 is the most common [78].

It has been shown that HPV-positive HNSCC cells are more radiosensitive with regard to their clonogenicity, because of an increased incidence of DNA double-strand breaks and simultaneously a defective DNA repair [79]. Kimple et al. postulated the enhanced induction of apoptosis as an explanation for the increased radiosensitivity of the HPV-driven HNSCC cells [80]. We observed that at clinically relevant early time points after RT, in both the HPV-negative and the HPV-positive tumor cells increased apoptosis was present, while necrotic tumor cells were not induced (Fig. 2b and c). Cell death behavior is directly related to phagocytosis since it is the task of phagocytosing cells to distinguish dying/dead cells from living ones and to eliminate them [81]. The so-called clearance of dying cells is generally regulated by a group of “eat me” signals, facilitating the cellular uptake by binding to phagocytic receptors on immune cells [82–85]. Living (tumor) cells, on the other hand, prevent phagocytotic clearance through the sustained presentation of “don’t eat me” surface signals, such as CD47 [86, 87]. Since one of the primary functions of macrophages and central mechanisms by which TAMs can affect cancer progression is through phagocytosis of pathogens and abnormal and dead cells including tumor cells [22, 62–64], phagocytosis is a therapeutic target for a wide range of clinical applications [65–67]. Similar to our study, Gardner et al. very recently developed an in vitro phagocytosis assay of M‑CSF-differentiated macrophages and irradiated brain tumor cells. They demonstrated that the phagocytosis of irradiated tumor cells was enhanced in a dose-dependent manner [88]. We now show for the first time that HNSCC cells are phagocytosed very well by M‑CFS-generated macrophages including the M2 phenotype, irrespective of the treatment and HPV status. (Fig. 3c and d). By contrast, GM-CSF-generated macrophages had lower phagocytosis rates. However, a significant increase in the uptake of irradiated HPV-positive cells by M0g macrophages when compared to HPV-negative cells was observed. This, together with the observation of decreased CD206 expression on M2 macrophages after contact with irradiated HPV-negative cells, provides the first indications of the impact of HPV status of HNSCC cells in the modulation of the macrophage response to irradiated tumor cells.

Of particular interest is that M1-MΦ phagocytosed irradiated HNSCC cells significantly better than non-irradiated ones. This might positively affect antitumor immune responses to RT-treated HNSCC as non-irradiated tumor cells should be quickly removed by M2-MΦ, whereas some of the irradiated ones could be phagocytosed by M1-MΦ. The latter are capable of initiating antitumor immune responses [89]. This could be mechanistically similar to the shift in the uptake of irradiated tumor cells from immune-suppressive macrophages to dendritic cells [90].

Although the prognostic relevance of macrophage polarization for tumor outcome is still debated [91, 92], it is, nevertheless, already extensively verified that there is a strong correlation of M2-MΦ with an increased tumor progression and a poorer overall prognosis for patients with HNSCC [46, 57, 70, 91, 93]. Because TAMs are considered as M2-like macrophages [58–60] and are therefore able to dampen antitumor immune responses by inducing an immunosuppressive environment, new strategies that directly target TAM polarization or re-polarization toward the M1 phenotype in TME represent an active area of research to improve antitumor therapies [94]. Several lines of evidence indicate the capability of macrophages to re-polarize from one phenotype to another depending on the microenvironment and on specific immunological mediators they are exposed to [95–98]. Porcheray et al., for example, demonstrated that macrophages stimulated toward a specific phenotype have the ability to return to a rather calm state after signal arrest, or to switch their activation phenotype rapidly upon counter stimulation [53]. However, in contrast to investigating the effect of changing the cytokine milieu surrounding the macrophages, in the present work the specific stimuli that the macrophages were exposed to were not only soluble factors of the SN, but additionally the tumor cells themselves.

Converting TAMs to a pro-inflammatory M1-like phenotype has become an attractive strategy for antitumor immunotherapy [99]. We now show for the first time that M1-MΦ are very stable in their phenotype irrespective of being in contact with non-irradiated or irradiated HPV-negative or HPV-positive HNSCC cells (Fig. 4a, b, e, f). Regarding the M2 phenotype, the incubation of macrophages with M‑CSF leads to an increase in the expression of the hemoglobin scavenger receptor CD163, which can be regarded as the best marker for M2-MΦ [40, 42, 48, 100], and by adding IL-10 its expression could be even more upregulated (Fig. 1f and g). Nevertheless, no changes in the CD163 expression were detected after adding the tumor cells to the macrophages, regardless of whether those cells were previously irradiated or left untreated (Fig. 4c and g). Due to the rather pro-tumoral TME in HNSCC [101], one could have suspected an increase in the expression of the M2 markers CD163 and CD206 after adding the tumor cells plus their corresponding SN to the unpolarized macrophages. On the contrary, a significant downregulation of the expression of the mannose receptor CD206 after the addition of irradiated HPV-negative (cal33) tumor cells was observed (Fig. 4d). This could contribute to reduced malignant progression in HNSCC [102] and should be examined in more detail according to the distinct radiation schedules for HNSCC in the future.

The development of ex vivo phagocytosis assays with macrophages of differing phenotypes, as they are present in vivo, is vital for preclinical screening of treatments of tumor cells in order to obtain evidence on the effects of the anticancer therapies [88]. We succeeded in developing such protocols for HNSCC and showed for the first time that HNSCC cells treated with hypofractionated irradiation have a better up-take by M1–MΦ, reduce CD206 on M2–MΦ compared to non-irradiated ones, and that HPV-positive irradiated cells are taken up better by M0g macrophages when compared to HPV-negative cells. Macrophage reprogramming will also need to be followed up more clinically in the future to provide more detailed information on the immunogenic effect of specific irradiation protocols in HNSCC.
